# Supplementary material for: Discovery and application of insertion-deletion (INDEL) polymorphisms for QTL mapping of early life-history traits in Atlantic salmon
Source: BMC Genomics. 2010 Mar 8;11:156. doi: 10.1186/1471-2164-11-156 (PMC2838853; doi:10.1186/1471-2164-11-156)
Supplement: Additional file 2 — Information on developed 76 locus single-run INDEL panel in Atlantic salmon. Information on fluorescence labeling, primer concentrations, PCR pooling and links to alignments, INDEL motifs and GENESCAN (Burge and Karlin 1997) predictions of genes/exons are available in html format. [file 1471-2164-11-156-S2.ZIP › Additionalfile2/snpsummary5134.html]

```
Cluster 1274 Contig 2

prev  Summary    Contig List  next
```

Size of Consensus sequence = 1773

Number of sequences = 38

Minimum redundancy = 6

Key

A gi|89876430|gb|DY732553.1|DY732553 EST\_ssal\_rgb2\_88292 ssalrgb2 mixed\_tissue Salmo salar cDNA Salmo salar cDNA clone ssal\_rgb2\_644\_140\_fwd 3', mRNA sequence  
B gi|117532949|gb|EG864394.1|EG864394 EST\_ssal\_eve\_57246 ssaleve thyroid Salmo salar cDNA Salmo salar cDNA clone ssal\_eve\_578\_003\_rev 5', mRNA sequence  
C gi|29312007|gb|CB500781.1|CB500781 ssalga506383 head Salmo salar cDNA, mRNA sequence  
D gi|117500009|gb|EG832138.1|EG832138 EST\_ssal\_eve\_44217 ssaleve thyroid Salmo salar cDNA Salmo salar cDNA clone ssal\_eve\_560\_043\_rev 5', mRNA sequence  
E gi|117500010|gb|EG832139.1|EG832139 EST\_ssal\_eve\_44218 ssaleve thyroid Salmo salar cDNA Salmo salar cDNA clone ssal\_eve\_560\_043\_fwd 3', mRNA sequence  
F gi|85023497|gb|DW552153.1|DW552153 EST\_ssal\_rgb2\_16572 rgb2 Salmo salar cDNA clone ssal\_rgb2\_527\_133\_fwd 3', mRNA sequence  
G gi|89834115|gb|DY694929.1|DY694929 EST\_ssal\_rgb2\_50668 ssalrgb2 mixed\_tissue Salmo salar cDNA Salmo salar cDNA clone ssal\_rgb2\_582\_037\_fwd 3', mRNA sequence  
H gi|89846161|gb|DY702284.1|DY702284 EST\_ssal\_rgb2\_58023 ssalrgb2 mixed\_tissue Salmo salar cDNA Salmo salar cDNA clone ssal\_rgb2\_593\_241\_fwd 3', mRNA sequence  
I gi|85028959|gb|DW557615.1|DW557615 EST\_ssal\_rgb2\_22034 rgb2 Salmo salar cDNA clone ssal\_rgb2\_535\_316\_fwd 3', mRNA sequence  
J gi|85050770|gb|DW578948.1|DW578948 EST\_ssal\_rgb2\_43367 rgb2 Salmo salar cDNA clone ssal\_rgb2\_570\_107\_fwd 3', mRNA sequence  
K gi|29321887|gb|CB510661.1|CB510661 ssalnwh506253 whole Salmo salar cDNA, mRNA sequence  
L gi|117528281|gb|EG859922.1|EG859922 EST\_ssal\_eve\_53220 ssaleve thyroid Salmo salar cDNA Salmo salar cDNA clone ssal\_eve\_572\_202\_rev 5', mRNA sequence  
M gi|117512461|gb|EG844220.1|EG844220 EST\_ssal\_eve\_37091 ssaleve thyroid Salmo salar cDNA Salmo salar cDNA clone ssal\_eve\_550\_188\_rev 5', mRNA sequence  
N gi|117512782|gb|EG844541.1|EG844541 EST\_ssal\_eve\_9957 ssaleve thyroid Salmo salar cDNA Salmo salar cDNA clone ssal\_eve\_513\_185\_rev 5', mRNA sequence  
O gi|117828060|gb|EG900756.1|EG900756 EST\_ssal\_evf\_39581 ssalevf mixed\_tissue Salmo salar cDNA Salmo salar cDNA clone ssal\_evf\_552\_314\_rev 5', mRNA sequence  
P gi|85049843|gb|DW578021.1|DW578021 EST\_ssal\_rgb2\_42440 rgb2 Salmo salar cDNA clone ssal\_rgb2\_568\_295\_fwd 3', mRNA sequence  
Q gi|117535593|gb|EG867038.1|EG867038 EST\_ssal\_eve\_28526 ssaleve thyroid Salmo salar cDNA Salmo salar cDNA clone ssal\_eve\_538\_293\_rev 5', mRNA sequence  
R gi|117506437|gb|EG838196.1|EG838196 EST\_ssal\_eve\_12669 ssaleve thyroid Salmo salar cDNA Salmo salar cDNA clone ssal\_eve\_517\_088\_rev 5', mRNA sequence  
S gi|117498635|gb|EG830852.1|EG830852 EST\_ssal\_eve\_43059 ssaleve thyroid Salmo salar cDNA Salmo salar cDNA clone ssal\_eve\_558\_214\_rev 5', mRNA sequence  
T gi|117498636|gb|EG830853.1|EG830853 EST\_ssal\_eve\_43060 ssaleve thyroid Salmo salar cDNA Salmo salar cDNA clone ssal\_eve\_558\_214\_fwd 3', mRNA sequence  
U gi|45306255|gb|CK876624.1|CK876624 SGP137908 Atlantic salmon Eye cDNA library Salmo salar cDNA clone OY4-0807 5', mRNA sequence  
V gi|117512459|gb|EG844218.1|EG844218 EST\_ssal\_eve\_37090 ssaleve thyroid Salmo salar cDNA Salmo salar cDNA clone ssal\_eve\_550\_188\_fwd 3', mRNA sequence  
W gi|89846162|gb|DY702285.1|DY702285 EST\_ssal\_rgb2\_58024 ssalrgb2 mixed\_tissue Salmo salar cDNA Salmo salar cDNA clone ssal\_rgb2\_593\_241\_rev 5', mRNA sequence  
X gi|45324666|gb|CK894933.1|CK894933 SGP160548 Atlantic salmon Spleen cDNA library Salmo salar cDNA clone MI5-1264 5', mRNA sequence  
Y gi|85023496|gb|DW552152.1|DW552152 EST\_ssal\_rgb2\_16571 rgb2 Salmo salar cDNA clone ssal\_rgb2\_527\_133\_rev 5', mRNA sequence  
Z gi|83641266|gb|DW007856.1|DW007856 SGP151766 Atlantic salmon Red muscle cDNA library Salmo salar cDNA clone RM6-0395 5', mRNA sequence  
a gi|89834113|gb|DY694928.1|DY694928 EST\_ssal\_rgb2\_50667 ssalrgb2 mixed\_tissue Salmo salar cDNA Salmo salar cDNA clone ssal\_rgb2\_582\_037\_rev 5', mRNA sequence  
b gi|117535592|gb|EG867037.1|EG867037 EST\_ssal\_eve\_28525 ssaleve thyroid Salmo salar cDNA Salmo salar cDNA clone ssal\_eve\_538\_293\_fwd 3', mRNA sequence  
c gi|117506438|gb|EG838197.1|EG838197 EST\_ssal\_eve\_12670 ssaleve thyroid Salmo salar cDNA Salmo salar cDNA clone ssal\_eve\_517\_088\_fwd 3', mRNA sequence  
d gi|84208190|gb|DW179915.1|DW179915 SGP303522 Atlantic salmon Gills II cDNA library Salmo salar cDNA clone SS0103C06 5', mRNA sequence  
e gi|117512771|gb|EG844530.1|EG844530 EST\_ssal\_eve\_9956 ssaleve thyroid Salmo salar cDNA Salmo salar cDNA clone ssal\_eve\_513\_185\_fwd 3', mRNA sequence  
f gi|117528278|gb|EG859919.1|EG859919 EST\_ssal\_eve\_53219 ssaleve thyroid Salmo salar cDNA Salmo salar cDNA clone ssal\_eve\_572\_202\_fwd 3', mRNA sequence  
g gi|117532950|gb|EG864395.1|EG864395 EST\_ssal\_eve\_57247 ssaleve thyroid Salmo salar cDNA Salmo salar cDNA clone ssal\_eve\_578\_003\_fwd 3', mRNA sequence  
h gi|85028958|gb|DW557614.1|DW557614 EST\_ssal\_rgb2\_22033 rgb2 Salmo salar cDNA clone ssal\_rgb2\_535\_316\_rev 5', mRNA sequence  
i gi|89876429|gb|DY732552.1|DY732552 EST\_ssal\_rgb2\_88291 ssalrgb2 mixed\_tissue Salmo salar cDNA Salmo salar cDNA clone ssal\_rgb2\_644\_140\_rev 5', mRNA sequence  
j gi|85050769|gb|DW578947.1|DW578947 EST\_ssal\_rgb2\_43366 rgb2 Salmo salar cDNA clone ssal\_rgb2\_570\_107\_rev 5', mRNA sequence  
k gi|85049844|gb|DW578022.1|DW578022 EST\_ssal\_rgb2\_42441 rgb2 Salmo salar cDNA clone ssal\_rgb2\_568\_295\_rev 5', mRNA sequence  
l gi|117828059|gb|EG900755.1|EG900755 EST\_ssal\_evf\_39580 ssalevf mixed\_tissue Salmo salar cDNA Salmo salar cDNA clone ssal\_evf\_552\_314\_fwd 3', mRNA sequence

6 SNPs detected

A B C D E F G H I J K L M N O P Q R S T U V W X Y Z a b c d e f g h i j k l  cosegregation weighted

421 T - - - - T - T T T - - - - - - - - - - T - . . . . . . . . . . . . . . . .   6/6 57.89
422 T - - - - T - T T T - - - - - - - - - - T - . . . . . . . . . . . . . . . .   6/6 57.89
423 C - - - - C - C C C - - - - - - - - - - C - . . . . . . . . . . . . . . . .   6/6 57.89
424 A - - - - A - A A A - - - - - - - - - - A - . . . . . . . . . . . . . . . .   6/6 57.89
425 C - - - - C - C C C - - - - - - - - - - C - . . . . . . . . . . . . . . . .   6/6 57.89
426 T - - - - T - T T T - - - - - - - - - - T - . . . . . . . . . . . . . . . .   6/6 57.89
